# Antimicrobial and Antibiofilm Peptides

**DOI:** 10.3390/biom10040652

**Published:** 2020-04-23

**Authors:** Angela Di Somma, Antonio Moretta, Carolina Canè, Arianna Cirillo, Angela Duilio

**Affiliations:** 1 Dipartimento di Scienze Chimiche, Università Federico II, 80126 Naples, Italy; angela.disomma@unina.it (A.D.S.); cane@ceinge.unina.it (C.C.); 2 Istituto Nazionale Biostrutture e Biosistemi (INBB), 00136 Rome, Italy; 3 Dipartimento di Scienze, Università degli Studi della Basilicata, 85100 Potenza, Italy; antonio.moretta@unibas.it; 4 CEINGE Biotecnologie Avanzate, 80145 Naples, Italy; cirilloa@ceinge.unina.it

**Keywords:** antimicrobial peptides, biofilm, mechanism of action, biofilm formation inhibition, resistance

## Abstract

The increasing onset of multidrug-resistant bacteria has propelled microbiology research towards antimicrobial peptides as new possible antibiotics from natural sources. Antimicrobial peptides are short peptides endowed with a broad range of activity against both Gram-positive and Gram-negative bacteria and are less prone to trigger resistance. Besides their activity against planktonic bacteria, many antimicrobial peptides also show antibiofilm activity. Biofilms are ubiquitous in nature, having the ability to adhere to virtually any surface, either biotic or abiotic, including medical devices, causing chronic infections that are difficult to eradicate. The biofilm matrix protects bacteria from hostile environments, thus contributing to the bacterial resistance to antimicrobial agents. Biofilms are very difficult to treat, with options restricted to the use of large doses of antibiotics or the removal of the infected device. Antimicrobial peptides could represent good candidates to develop new antibiofilm drugs as they can act at different stages of biofilm formation, on disparate molecular targets and with various mechanisms of action. These include inhibition of biofilm formation and adhesion, downregulation of quorum sensing factors, and disruption of the pre-formed biofilm. This review focuses on the proprieties of antimicrobial and antibiofilm peptides, with a particular emphasis on their mechanism of action, reporting several examples of peptides that over time have been shown to have activity against biofilm.

## 1. Introduction

In 1922, Alexander Fleming identified lysozyme from nasal mucus [1], which was considered the first human antimicrobial protein. This discovery was overshadowed when in 1928, Fleming discovered penicillin, which, together with streptomycin, in 1943, led to the beginning of the so-called “Golden Age of Antibiotics”. In the 1940s, along with Howard Florey and Ernst Chain, he brought the therapeutic use of penicillin to fruition, which allowed these scientists to be awarded the Nobel Prize for Medicine in 1945.

**Figure 1:**
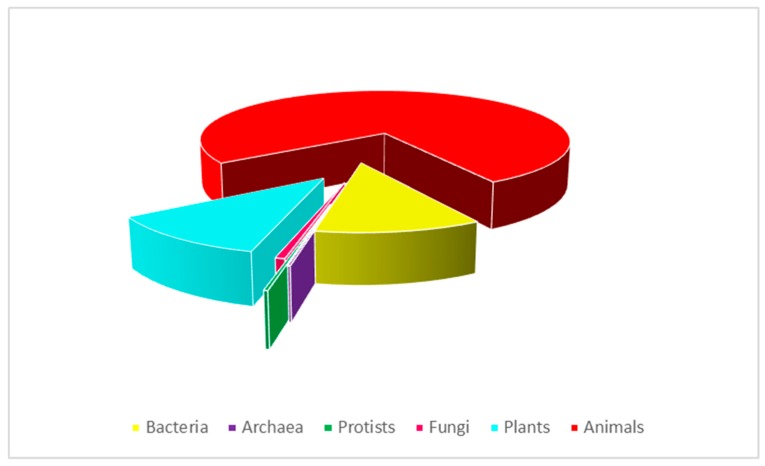
Antimicrobial peptides from the Antimicrobial Peptide Database (total of 3180). Data updated to 10th April 2020.

With the advent of the “Golden Age of Antibiotics”, there was a loss of interest in the therapeutic potential of natural antimicrobial peptides (AMPs), such as lysozyme [2,3]. However, in the 1960s, due to the increase in the number of multidrug-resistant microbial pathogens, the attention of the scientific community turned to the study of antimicrobial peptides [4,5,6,7]. Antimicrobial peptides are small molecules (10–100 amino acids) produced by all living organisms that play an essential role in the innate immunity [8,9]. Since the discovery of the first groups of AMPs, the magainins from the skin of the African clawed frog *Xenopus laevis* by Zasloff et al. [10,11,12] and the first antimicrobial peptides isolated from the insect *Hyalophora cecropia* by Boman [13], an ever-increasing number of AMPs have been identified and studied. The Antimicrobial Peptide Database (APD, http://aps.unmc.edu/AP), which is constantly updated, contains 3180 antimicrobial peptides from 6 kingdoms: 355 from bacteria, 5 from archaea, 8 from protists, 20 from fungi, 352 from plants, and 2356 from animals, including some synthetic peptides (Figure 1). Cationic AMPs are the largest group even if anionic peptides have also been identified in vertebrates, invertebrates, and plants [9]. Antimicrobial peptides show a broad range of activity against Gram-negative and Gram-positive bacteria, fungi, mycobacteria, and some enveloped viruses [11]. In addition, it has been shown that they might also have cytotoxic effects against cancer cells [14,15,16].

A further aspect of the AMPs activity that has been much investigated in recent years and needs to be more deeply considered is their ability to affect biofilm formation. Biofilms are a complex ensemble of microbial cells irreversibly associated to surfaces and enclosed in an essentially self-produced matrix consisting of polysaccharides, DNA, and proteins. They are ubiquitous in nature, having the ability to adhere to virtually any surface, either biotic or abiotic, including medical devices, causing chronic infections that are difficult to eradicate [17]. The biofilm matrix plays an active role in the development of antimicrobial resistance, protecting bacteria from the host immune system, hostile environmental conditions, and antimicrobial agents, including the majority of antibiotics. Biofilms are very difficult to treat due to their adaptive resistance to antibiotics compared to their planktonic counterparts [17]. Many AMPs show antibiofilm activity against multidrug-resistant bacteria, acting at different stages of biofilm formation, on disparate molecular targets and with various mechanisms.

This review focuses on antimicrobial peptides and their mechanism of action against biofilm formation.

## 2. Antimicrobial Peptides

### 2.1. Structure

**Figure 2:**
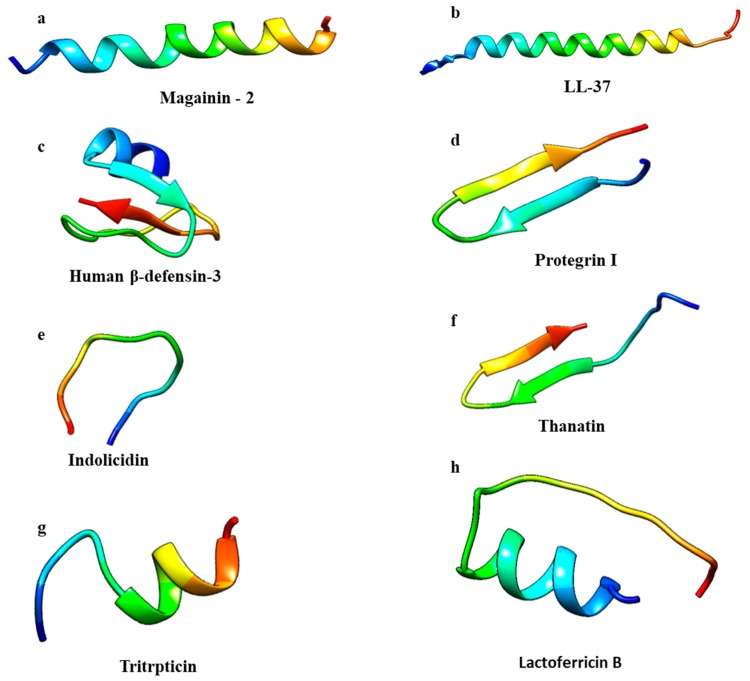
Antimicrobial peptide classes: α-helical, β-sheet, loop, and extended. Structures were generated by CHIMERA software [31]. PDB codes: (**a**) 2MAG, Magainin-2; (**b**) 2K6O, LL-37; (**c**) 1KJ5, Human β-defensin-3; (**d**) 1PG1, Protegrin I; (**e**) 1G89, Indolicidin; (**f**) 5XO3, Thanatin; (**g**) 1D6X, Tritrpticin; (**h**) 1LFC, Lactoferricin B.

AMPs can be classified in four groups according to their secondary structure: α-helical, β-sheet, loop, and extended peptides [18]. α-helical and β-sheet peptides are more common and AMPs endowed with α-helical structures are the most studied to date [19]. α-helical AMPs are linear in aqueous solution and will assume amphipathic helical structures when they interact with bacterial membranes or in the presence of organic solvents [6]. Magainin-2 and LL-37 are examples of peptides that belong to this group (Figure 2a,b) [20,21]. In the α-helix conformation, the distance between two close amino acids is around 0.15 nm while the angle between them with regard to the center is around 100 degrees from the top view [18].

β-sheet peptides are stabilized by at least two disulphide bridges, organized to create an amphipathic structure [19,22,23]. This class includes protegrins (from the cathelicidin family); defensins, the largest group of β-sheet AMPs; and tachyplesins (Figure 2c,d) [24,25]. Due to their rigid structure, β-sheet AMPs are more structured in solution and do not undergo major conformational changes when interacting with a membrane environment [26,27]. Thanatin and lactoferricin B are peptides with a loop structure, stabilized by disulfide, amide, or isopeptide bonds (Figure 2e,f) [19].

The extended AMPs class is populated by peptides that do not show a regular secondary structure. These peptides are rich in arginine, tryptophan, glycine, proline, and histidine residues [19,28]. The 13-residue Arg- and Trp-rich tritrpticin and indolicidin peptides (Figure 2g,h) from porcine and bovine leukocytes, respectively, belong to this group of AMPs [29]. Due to their short length, a simple residue substitution can lead to broad changes in both their structural and functional properties. As an example, replacing Pro residues with Ala in tritrpticin will transform the peptide structure into an α-helical conformation with improved antimicrobial activity but also with higher cytotoxicity [30].

Antimicrobial peptides have a wide spectrum of action against bacteria, viruses, cancer cells, fungi, and parasites [11,14] as described in the following sections.

### 2.2. Antibacterial Peptides

Antibacterial peptides are among the most studied and are characterized by both hydrophobic and hydrophilic domains. Most of them are cationic and this positive net charge allows these peptides to interact with the negatively charged bacterial membranes [32]. Their mechanism of action has been widely studied. AMPs can lead to bacterial cell death through both membranolytic [33,34,35] and non-membranolytic mechanisms, interacting with intracellular targets, such as DNA, RNA, and proteins [36,37,38,39]. Both Gram-negative and Gram-positive bacteria have molecules on the outer membrane that confer a negative net charge, allowing the electrostatic interaction with cationic peptides [24]. Then, the AMPs accumulate at the surface and, once a certain concentration is reached, they assemble on the bacterial membrane [40].

Three different putative models have been proposed to describe the action of antimicrobial peptides. In the barrel-stave model, peptides perpendicularly insert into the membrane, promoting peptide–peptide lateral interactions. In this mechanism, the AMPs’ amphipathic structure plays a significant role because the hydrophilic residues generate the channels’ lumen while the hydrophobic side establishes a favorable interaction with membrane lipids [41]. To date, only a few peptides, such as pardaxin and alamethicin, that act through this mechanism have been identified [42,43].

The same event of peptide insertion into the membrane occurs in the toroidal model although the pore formation is not originated by peptide–peptide interactions. In this model, the peptide induces a curvature in the lipid bilayer and the pore is generated by both the peptide and the phospholipid head groups [44]. The essential difference between these two models is the arrangement of the lipid bilayer, as in the toroidal model, the hydrophobic and hydrophilic arrangement of the bilayer is disrupted while it is intact in the barrel-stave model. Many AMPs acting in the toroidal model have been found, including magainin-2 [25], protegrin-1 [45], melittin [46], and lacticin Q [25].

In the carpet model, the AMPs adsorb onto the membrane, covering the entire surface until a threshold concentration is reached [26]. At this stage, a detergent-like effect occurs, leading to the loss of membrane integrity and eventually to disintegration by micelle formation. In this model, specific peptide–peptide interactions are not required, and peptides do not insert into the hydrophobic core to form transmembrane channels [26]. Antimicrobial peptides like LL-37 and cecropin are known to adopt the carpet model mechanism [47,48].

In the non-membranolytic mechanism, peptides can inhibit cell wall and protein synthesis, bacterial cell division, or DNA replication by interacting with specific proteins involved in this biological process. As an example, Di Somma et al. [49] demonstrated that temporin-L (TL) interacts with *E. coli* FtsZ, a protein belonging to the divisome complex, leading to inhibition of the Z-ring formation, thus impairing cell division and causing bacterial death without damaging the cell membrane. Graf et al. reported the subclass of proline-rich AMPs (PrAMPs) that can penetrate the bacterial membrane and kill bacteria by inhibiting protein synthesis [39]. In particular, Mardirossian et al. tested the antimicrobial activity of Bac5 (1–25), an N-terminal fragment of the bovine proline-rich antimicrobial peptide Bac5, on *Escherichia coli, Acinetobacter baumannii, Klebsiella pneumoniae, Staphylococcus aureus, Salmonella enterica*, and *Pseudomonas aeruginosa*, showing the inhibition of bacterial protein synthesis [40]. In addition, the synthetic peptide 35409 has been reported to inhibit cell division and induce filamentation, suggesting two different targets within a bacterial cell [41], or the lysine-peptoid hybrid, LP5, binds DNA gyrase and topoisomerase IV, causing inhibition of thee replication and ATP leakage from bacterial cells [42].

### 2.3. Anticancer Peptides

Antimicrobial peptides with anticancer activity, also called anticancer peptides (ACPs), are α-helical or β-sheet peptides and can be divided into two groups. Peptides, such as insect cecropins and frog skin magainins, belong to the first group, characterized by peptides active against both bacteria and cancer cells but not against normal mammalian cells [50,51,52]. Peptides toxic to bacteria and both normal and cancer cells, including the bee venom melittin, insect defensins, and the human LL-37 peptide [53,54], belong to the second group. ACPs can lead to cancer cells’ death by membranolytic or non-membranolytic mechanisms according to the peptide characteristics and the peculiar target membrane features [55]. Cancer cells differ from normal mammalian cells due to their membrane net negative charge, which is conferred by anionic molecules, such as the phospholipids phosphatidylserine (PS), heparin sulfate, O-glycosylated mucins, and sialylated gangliosides. Differently, mammalian cell membranes are endowed with a zwitterionic character due to the molecules normally present on their membranes [14,45]. In healthy cells, the phosphatidylserine molecules are in the plasma membrane’s inner-leaflet, while in cancer cells, the asymmetry between inner and outer membrane leaflets is lost, leading to the presence of PS in the outer leaflet [56,57]. The negative net charge exposed on the cancer outer membrane makes them similar to the bacterial membranes, suggesting that AMPs and ACPs might share similar molecular principles for selectivity and activity [58]. Dermaseptin B2 and B3 have been reported to be active against the proliferation of human prostate, mammary, and lymphoma cancer cells [58]. A study conducted by Lin et al. on the cytotoxic effect of epinecidin-1 on normal and cancer cells showed that this peptide could inhibit the growth of both tumor and normal cell lines. It was also demonstrated that epinecidin-1 induces cytotoxic effects and membrane lysis, perturbating the cancer cell membrane. In addition, this peptide inhibits necrosis in HT1080 cells (highly aggressive fibrosarcoma cell line) by downregulating the necrosis-related genes [59].

### 2.4. Antiviral Peptides

Because of the emerging resistance of viruses and the limited efficiency of commonly used drugs, antiviral peptides represent good candidates as putative therapeutic agents [60]. Antiviral agents can act at different stages, by inhibiting the activity of viral reverse transcriptase or the pre-integration complex or avoiding the transport of circular viral DNA to the nucleus. Alternatively, they can inhibit the action of viral integrase, impairing viral DNA to integrate into the cellular chromosome. In addition, antiviral compounds may inhibit the viral proteases by blocking the retroviral morphogenesis because, after transcription, the proviral DNA is translated into a polyprotein that requires the activity of viral proteases to generate the proteins needed to assemble the viral capsid [61].

It has been demonstrated that both enveloped RNA and DNA viruses can be targeted by antiviral peptides [62]. AMPs can cause membrane instability by integrating into viral envelopes, thus preventing the viruses from infecting host cells [63]. Melittin, in addition to anticancer activity, has also been reported to have inhibitory activity against enveloped viruses, such as Junin virus (JV), HIV-1, and HSV-2. Melittin was suggested to suppress HSV-1 syncytial mutant-mediated cell fusion, very likely by interfering with the activity of Na+ K+ ATPase, a cellular enzyme involved in the membrane fusion process [64]. Some antiviral AMPs can prevent viral particles from entering the host cells by binding specific receptors on mammalian cells. For example, some α-helical cationic peptides, such as lactoferrin, can prevent HSV infections by binding to heparan sulfate molecules needed for the attachment of HSV viral particles to the host cell surface, thus blocking virus–receptor interactions [65,66].

### 2.5. Antifungal Peptides

According to their mechanism of action and origin, antifungal peptides can be grouped into membrane-traversing peptides, which can lead to pore formation or act on β-glucan or chitin synthesis, and non-membrane-traversing peptides that interact with the cell membrane and cause cell lysis [67]. Antifungal peptides can lead to fungi death through different mechanisms of action, including inhibition of DNA, RNA, and protein synthesis; induction of apoptotic mechanisms; permeabilization of membranes; inhibition of cell wall synthesis and enzyme activity; or repression of protein folding and metabolic turnover [68,69].

### 2.6. Antiparasitic Peptides

Magainins and cecropins were the first identified antimicrobial peptides that exhibited antiparasitic activity [70]. Although some parasitic microorganisms are multicellular, the mechanism of action of antiparasitic peptides (APPs) is very similar to AMPs, directly interacting with the cell membrane [71]. Scorpine, a peptide deriving from the venom of the scorpion *Pandinus imperator*, is able to inhibit the developmental stages of both the ookinete and gamete of *Plasmodium berghei* [72]. Bombinin H4 was reported to affect the viability of both insect and mammalian forms of *Leishmania* through perturbation of the plasma membranes at micromolar concentrations. The molecular mechanism consists in a rapid depolarization of the plasma membrane and loss of integrity associated with bioenergetic collapse [73]. Cathelicidin is a further example of APP that is able to kill *Caernohabditis elegans* through pore formation on the cell membrane [74].

## 3. Biofilm

Biofilm consists of a mixture of microorganisms embedded in self-produced extracellular polymeric substances (EPSs). The EPS constitutes a structural scaffold for other carbohydrates, proteins, nucleic acids, and lipids to adhere to. The presence of biofilms represents a severe problem in environmental, food, and biomedical fields as these architectures protect bacteria from hostile environments and prevent the effect of antimicrobial agents [75]. The exopolysaccharides’ characteristics differ among various bacteria and depend on the growth conditions, medium, and availability of nutrients. In some forms of biofilm, mannose, galactose, and glucose are the most abundant carbohydrates, followed by N-acetyl-glucosamine, galacturonic acid, arabinose, fucose, rhamnose, and xylose, which occurr in the composition of the biofilm matrix from *Enterococcus faecalis*, *Staphylococcus aureus*, *Klebsiella pneumoniae*, *Acinetobacter baumannii*, and *Pseudomonas aeruginosa* [76]. Most exopolysaccharides are not biofilm specific, but their production increases following a stress response, such as the production of colanic acid in *Escherichia coli* and the alginate synthesis in *P. aeruginosa* [77].

Biofilm formation and development consist of four different stages: (i) Aggregation or attachment; (ii) microbe adhesion; (iii) biofilm development and maturity; and (iiii) biofilm aging [78]. The aggregation or attachment step is divided into a reversible and irreversible phase. The reversible adhesion begins when the microorganisms come in contact with the target surface. During this event, some weak interactions, including van der Walls and electrostatic forces, and hydrophobic interactions between the molecules occurring on microbial cells and those present on the target surface are established. Afterwards, the irreversible adhesion phase takes place with the formation of covalent interactions and the initial production of exopolysaccharides. In the adhesion step, the formed microcolonies are protected by extracellular polysaccharides or by cellular organelles, such as pili and fimbriae, that allow bacterial cells to survive. During the third stage, the colony grows, acquiring a fungi-like architecture, and cells undergo further adaptation to life in a biofilm. In particular, two properties are often associated with surface-attached bacteria: The increased synthesis of EPSs and the development of antibiotic resistance. These features appear to create a protective environment and cause biofilms to be a tenacious clinical problem. Finally, in the last stage, the biofilm is capable of releasing part of the colonies into the environment and bacterial cells move to further colonize other surfaces in appropriate conditions, thus entering another biofilm cycle. Each stage of the biofilm formation process depends on the microbial genera and species, the characteristics of the attachment surface, the environmental conditions, and the physiological status of the microorganism [79]. Microorganisms’ attachment occurs more commonly on surfaces that are hydrophobic, rough, and coated by conditioning films. On the contrary, attachment to surfaces is made more complicated by electrostatic repulsion between the negative organic molecules of surfaces and the bacteria membrane.

### 3.1. Antimicrobial Peptides and Biofilm

The antibiofilm activity of antimicrobial peptides has been less studied than their antimicroorganism capabilities. Moreover, the assessment of a specific ability to impair biofilm formation well apart from their antimicrobial activity is quite difficult to achieve. An AMP can be considered to be antibiofilm if the minimum biofilm inhibitory concentration (MBIC) is below the minimum inhibitory concentration (MIC), with a distinct activity compared to the direct killing antimicrobial capability. Eradication of preformed biofilms is much more difficult than inhibition [80], and the minimum biofilm eradication concentration (MBEC), i.e., the minimum concentration of an antimicrobial agent required to eliminate pre-formed biofilms, is generally larger than MBIC. In all cases, it is fundamental to being able to distinguish between dead and living cells. Recently, Raheem and Straus [81] described many biological assays and biophysical methods and techniques to define the specific antibacterial and antibiofilm functions’ peptides. For all these reasons, few peptides endowed with real antibiofilm activity have been identified so far; some of these peptides are listed in Table 1.

**Figure 3:**
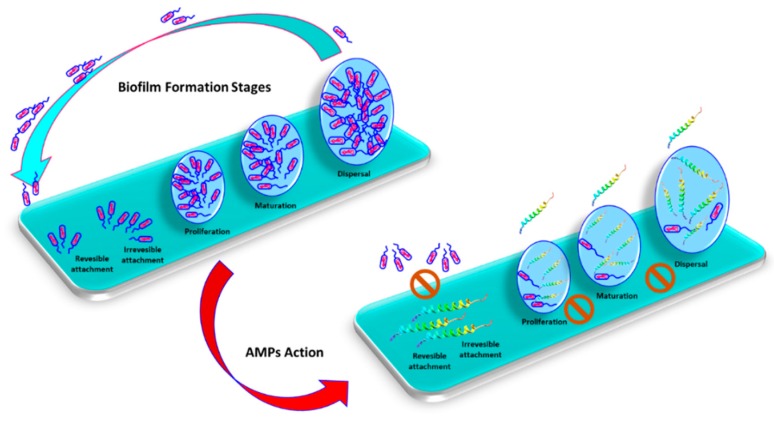
Biofilm formation consists on attachment, proliferation, mutation and detachment stages, which can be inhibited by antimicrobial peptides

Antibiofilm peptides were demonstrated to affect biofilm formation or degradation at different stages and with different mechanisms of action, including inhibition of biofilm formation and adhesion, downregulation of quorum sensing, and killing of pre-formed biofilm [88,89] (Figure 3).

Nisin A is able to disrupt or degrade the membrane of biofilm-embedded cells of an MRSA strain of *S. aureus,* disturbing the membrane potential [90]. Human cathelicidin LL-37, one of the most studied antibiofilm peptides, is able to affect the bacterial cell signaling system. This peptide can inhibit *P. aeruginosa* biofilm formation at a concentration of 0.5 μg/mL by downregulating the genes related to the QS system, decreasing the attachment of bacterial cells on the surface and stimulating twitching motility mediated by type IV pili [89,91].

Antimicrobial peptides can also lead to the degradation of the extracellular polymeric matrix of bacterial biofilms. Hepcidin 20 can reduce the extracellular matrix mass of *Staphylococcus epidermidis* and alter its biofilm architecture by targeting the polysaccharide intercellular adhesin (PIA) [92]. Antibiofilm peptides can also target a stringent stress response in both Gram-negative and Gram-positive bacteria or downregulate genes involved in biofilm formation and the transportation of binding proteins [93]. Biofilm formation in staphylococci depends on the synthesis of the polysaccharide intracellular adhesin (PIA), which is encoded by the icaADBC locus. Human β-defensin 3 was shown to be able to reduce the expression of the icaA, IcaR, and icaD genes of *S. epidermidis* ATCC 35,984, leading to a reduction of biofilm formation [94].

Gopal et al. [95] reported that NRC-16, a pleurocidin peptide analogue, showed MIC values ranging from 2.17 to 17.4 μg/mL on planktonic bacteria vs. biofilms against different Gram-negative and Gram-positive bacteria, and fungi. It is interesting to note that similar results were obtained with the melittin peptide. For both of them, minimal biofilm inhibitory concentration (MBIC) values ranging from 8 to 35 μg/mL against five clinical strains of *P. aeruginosa* have been obtained [95]. Moreover, Blower et al. [86] demonstrated that the SMAP-29 peptide is able to inhibit biofilm production in *Burkholderia thailandensis* by about 50% at peptide concentrations at or above 3 μg/mL. Anunthawan et al. studied KT2 and RT2, two synthetic tryptophan-rich cationic peptides, which showed activity against multidrug-resistant *E. coli* biofilms at sub-MIC levels [96]. Another peptide known as CRAMP is able to inhibit fungal biofilm formation [97], but surprisingly, it was demonstrated that AS10, a CRAMP shorter fragment, was able to inhibit biofilm growth of *Candida albicans*, *E. coli*, and *P. aeruginosa* [98]. Moreover, IDR-1018 showed antibiofilm activity against several Gram-positive and Gram-negative pathogens [99]. De la Fuente-Núñez et al. studied two synthetic peptides DJK-5 and DJK-6 based on properties associated with IDR-1018, which showed a broad spectrum of antibiofilm activity and the ability to eradicate pre-existing biofilms [100]. Mataraci and Dosler designed the CAMA peptide, a hybrid peptide (cecropin (1-7)–melittin A (2-9) amide) containing the N-terminal region of cecropin A and the N-terminal portion of melittin A. Interestingly, this peptide was able to inhibit methicillin-resistant *Staphylococcus aureus* (MRSA) biofilm formation [101].

### 3.2. Biofilm Resistance to Antimicrobial Peptides

One of the ideas associated to the biofilm resistance to AMPs is related to their interaction with EPS even if the mechanism is still not well understood. Most of the molecules making up EPS have a negative charge, but the exopolymer PIA, composed of poly-N-acetyl glucosamine, is positively charged and it might protect the biofilm from AMPs through electrostatic repulsion with the positively charged peptides [102]. In fact, PIA was demonstrated to defend *S. epidermidis* and *S. aureus* from the LL-37 and the human β-defensin peptides’ action [103].

Alginate, made up of the uronic acid D-mannuronate and the C-5 epimer-L guluronate, is an anionic extracellular polysaccharide secreted by Gram-negative bacteria that can interact with positively charged peptides, protecting biofilm-embedded cells. Alginate is able to trap antimicrobial peptides in hydrophobic microdomains consisting of pyranosyl C–H groups, which are inducible when the complexes AMPs-alginate are formed, owing to the charge neutralization between the two species [104,105].

In Gram-positive bacteria, the resistance to AMPs can be mediated by the membrane protein MprF, which is involved in the addition of alanine or lysine to phosphatidylglycerol (PG) to form alanyl-phosphatidylglycerol (APG) and lysyl-phosphatidylglycerol (LPG), respectively, and in the translocation of these compounds to the outer leaflet [106,107]. It was demonstrated that MprF mutants of *S. aureus* were more susceptible to AMPs, suggesting that the addition of lysine or arginine to the membrane could lead to a reduction in the susceptibility to AMPs [108]. An MprF homolog has been found in *P. aeruginosa* involved in the addition of alanine to PG to form APG. This modification led to an increased resistance to antimicrobial agents [109].

In *P. aeruginosa* and *Salmonella enterica*, the PhoP/PhoQ genetic system is able to decrease the LPS net negative charge by adding aminoarabinose to the lipid, conferring AMPs’ resistance to bacterial biofilms [110]. In *P. aeruginosa*, a two-component regulatory system pmrA-pmrB has also been found, which regulates resistance to LL-37, polymyxin B, and polymyxin E. This system modifies LPSs in the bacteria’s outer membrane, leading to a reduction of the AMPs’ interaction with the outer membrane [111]. Moreover, it was found that the addition of an acyl chain to lipid A might contribute to bacterial resistance to AMPs. In *S. enterica Typhimurium*, the PagP enzyme adds additional palmitate (C16:0 acyl chain) to the lipid A moiety. This acylation is thought to be responsible for the increase of the hydrophobic interactions between lipid A and the acyl chains, thus leading to a higher outer membrane fluidity [112]. A higher membrane permeability in response to AMPs was observed in pagP mutants of *S. enterica Typhimurium*, compared to the control strain [113]. It was also demonstrated that deacylation could increase the bacteria’s susceptibility to AMPs, thus supporting the finding that lipid A acylation is involved in the bacterial resistance to antimicrobial peptides. The PagL enzyme, located on the outer membrane of several Gram-negative bacteria, is responsible for the deacylation of lipid A, removing R-3-hydroxymyristate from position 3 of some lipid A precursor [114].

The two component systems (TCSs) are used by bacteria to respond to environmental changes. TCSs consist of a membrane sensor, which is able to detect signals from the environment that are transferred by activating a transcriptional response regulator through phosphorylation or de-phosphorylation. The receptor is usually a histidine kinase located in the cytoplasmic membrane that can be activated by environmental signals. The cytoplasmic protein is phosphorylated by the sensor and acts as a transcription factor. The response involves the activation of genes, such as membrane-remodeling genes, ion transporters, and virulence genes, which help Gram-negative and Gram-positive bacteria to better adapt to the environment. Several TCSs systems are known to respond to AMPs, thus helping bacteria to counteract their activity [115,116,117].

## 4. Discussion and Future Considerations

The identification of new therapeutic strategies to counteract biofilm-associated infections is among the main challenges in medicine. The high concentrations of antibiotics used in order to disrupt or prevent biofilm formation could be associated with poor prognosis and cytotoxicity. For this reason, a promising strategy might consist in the use of alternative drugs to address biofilm-related infections. Because of their peculiar characteristics, antimicrobial peptides have to be considered as valid candidates in the fight against biofilms. However, AMPs’ interaction with EPS components might affect their antimicrobial activity, representing an obstacle for the development of AMPs as antibiofilm drugs. Designed antibiofilm peptides could be used to interfere with signaling pathways involved in the synthesis of EPS components. Otherwise, EPS–AMP interactions could even be used for the design of AMP-based antibiofilm strategies in order to seize essential EPS components, interfering with the biofilm architecture.

The strategy of combining biofilm dispersing agents with conventional antibiotics could also be exploited. Bacterial invasions are often impossible to eradicate by the direct administration of antibiotics due to the protection effect exerted by biofilms, and the use of a high concentration of antibiotics has to be discouraged due to their extreme toxicity. AMP–AMP and AMP–drug combinations induce biofilm matrix degradation, allowing the antibacterial agent to escape protection and to reach bacterial cells, which may be potential areas of future anti-biofilm study and research. Promising combinatorial strategies can then be foreseen consisting in the use of AMPs with compounds able to dissolve the biofilm matrix or antimicrobial peptides in association with drugs used for anti-infective therapy, with anti-inflammatory ormucolytic agents, such as salicylic acid or ibuprofen, or with inhibitors of QS [118].

## Figures and Tables

**Table 1:** Some of the known antibiofilm peptides. Peptide name, sequence, and source are reported.

Peptide	Sequence	Source	Reference
Protegrin 1	RGGRLCYCRRRFCVCVGR	leukocytes; Pig, *Sus scrofa*	[82]
Pleurocidin	GWGSFFKKAAHVGKHVGKAALTHYL	skin mucous secretions, Winter flounder, *Pleuronectes americanus*	[83]
LL-37	LLGDFFRKSKEKIGKEFKRIVQRIKDFLRNLVPRTES	neutrophils, monocytes; mast cells; lymphocytes, Mesenchymal Stem Cells; islets; skin, sweat; airway surface liquid, saliva; *Homo sapiens*; Also *Pan troglodytes*	[84]
Indolicidin	ILPWKWPWWPWRR	bovine neutrophils, *Bos taurus*	[85]
SMAP-29	RGLRRLGRKIAHGVKKYGPTVLRIIRIAG	sheep leukocytes; *Ovis aries*	[86]
Human β defensin 3	GIINTLQKYYCRVRGGRCAVLSCLPKEEQIGKCSTRGRKCCRRKK	skin, tonsils, oral/saliva, *Homo sapiens*	[87]

## Abbreviations

AMPs	Antimicrobial peptides
APD	Antimicrobial Peptide Database
ACPs	Anticancer peptides
MBIC	Minimum Biofilm Inhibitory Concentration
MBEC	Minimum Biofilm Eradication Concentration
MIC	Minimum Inhibitory Concentration
QS	Quorum Sensing
PIA	Polysaccharide Intracellular Adhesin
EPS	Extracellular Polymeric Substances
PS	Phosphatidylserine
PG	Phosphatidylglycerol
APG	Alanyl - Phosphatidylglycerol
LPG	Lysyl - Phosphatidylglycerol
TCS	Two Component Systems
